# Neprilysin Inhibitors and Bradykinin

**DOI:** 10.3389/fmed.2018.00257

**Published:** 2018-09-19

**Authors:** Duncan J. Campbell

**Affiliations:** ^1^Department of Molecular Cardiology, St. Vincent's Institute of Medical Research, Fitzroy, VIC, Australia; ^2^Department of Medicine, The University of Melbourne, Parkville, VIC, Australia; ^3^St. Vincent's Hospital, Melbourne, VIC, Australia

**Keywords:** neprilysin, bradykinin, neprilysin inhibition, angioedema, ARNI

## Abstract

Bradykinin has important physiological actions related to the regulation of blood vessel tone and renal function, and protection from ischemia reperfusion injury. However, bradykinin also contributes to pathological states such as angioedema and inflammation. Bradykinin is metabolized by many different peptidases that play a major role in the control of bradykinin levels. Peptidase inhibitor therapies such as angiotensin converting enzyme (ACE) and neprilysin inhibitors increase bradykinin levels, and the challenge for such therapies is to achieve the beneficial cardiovascular and renal effects without the adverse consequences such as angioedema that may result from increased bradykinin levels. Neprilysin also metabolizes natriuretic peptides. However, despite the potential therapeutic benefit of increased natriuretic peptide and bradykinin levels, neprilysin inhibitor therapy has only modest efficacy in essential hypertension and heart failure. Initial attempts to combine neprilysin inhibition with inhibition of the renin angiotensin system led to the development of omapatrilat, a drug that combines ACE and neprilysin inhibition. However, omapatrilat produced an unacceptably high incidence of angioedema in patients with hypertension (2.17%) in comparison with the ACE inhibitor enalapril (0.68%), although angioedema incidence was less in patients with heart failure with reduced ejection fraction (HFrEF) treated with omapatrilat (0.8%), and not different from that for enalapril therapy (0.5%). More recently, LCZ696, a drug that combines angiotensin receptor blockade and neprilysin inhibition, was approved for the treatment of HFrEF. The approval of LCZ696 therapy for HFrEF represents the first approval of long-term neprilysin inhibitor administration. While angioedema incidence was acceptably low in HFrEF patients receiving LCZ696 therapy (0.45%), it remains to be seen whether LCZ696 therapy for other conditions such as hypertension is also accompanied by an acceptable incidence of angioedema.

## Introduction

Despite decreasing incidence, cardiovascular disease remains a major cause of premature morbidity and mortality (1), and there is a continuing search for new therapies for its prevention and treatment. LCZ696 (Entresto) is the first of a new drug class referred to as ARNI (dual acting angiotensin receptor-neprilysin inhibitor) that contains equimolar amounts of valsartan, a type 1 angiotensin II receptor blocker (ARB) and sacubitril, a prodrug that is hydrolyzed to form LBQ657, a potent inhibitor of neprilysin (Table 1). The approval of LCZ696 as therapy for heart failure with reduced ejection fraction (HFrEF) represents the first approval of long-term neprilysin inhibitor therapy. Neprilysin is a key enzyme in the degradation of natriuretic peptides, and the primary rationale for neprilysin inhibitor therapy in cardiovascular disease was to increase endogenous natriuretic peptide levels, and thereby achieve the vasodilatation and natriuresis these peptides produce. However, neprilysin degrades many other peptides, including bradykinin (17). Bradykinin may contribute not only to the benefits of neprilysin inhibitor therapy but also to the adverse effects of this therapy. Of particular concern for drugs that inhibit bradykinin degradation and thereby increase bradykinin levels is the risk of angioedema, with increased bradykinin levels implicated in both hereditary and drug-induced forms of angioedema (18–21). This review will briefly describe neprilysin, the kallikrein kinin system, and the role of neprilysin in bradykinin metabolism, and then discuss the potential role of kinins in mediating the therapeutic benefits and adverse effects of neprilysin inhibitor therapy.

**Table 1:** Specificity of neprilysin inhibitors (K_i_ or IC_50_).

**Inhibitor (units for K_i_ or IC_50_)**	**Neprilysin**	**NEP2**	**ACE**	**ECE-1**	**ECE-2**	**APP**	**References**
Thiorphan (nmol/L)	4, 4.7	120, 129, 250	150	No	>10 μmol/L^#^	>100 μmol/L	(2–6)
Phosphoramidon (nmol/L)	1.5, 2	0.8, 1.0	2	680^*^, 675^*^	1.2^#^, 4^#^	>10 μmol/L	(2, 3, 5–9)
Candoxatrilat (nmol/L)	3.2, 9.5	44	>10 μmol/L	6.5^†^		>10 μmol/L	(10, 11)
Omapatrilat (nmol/L)	0.45, ~2, 3, 5–8	8, 17, 25	0.64, 0.98, 5	10 μmol/L^†^		194, 250, 260	(3, 10–13)
LBQ657 (nmol/L)	2.3, 5	Yes	Yes, >10 μmol/L	No	Yes	No	(14–16)

*Inhibitor concentrations are shown as nmol/L, except where indicated to be μmol/L*.

* *pH: 7.2*;

† *pH 6.5*;

# *pH 5.5*.

*ACE, angiotensin converting enzyme; APP, aminopeptidase P; ECE-1, endothelin converting enzyme 1; ECE-2, endothelin converting enzyme 2; NEP2, neprilysin homolog membrane metalloendopeptidase-like 1*.

## Neprilysin

**Figure 1:**
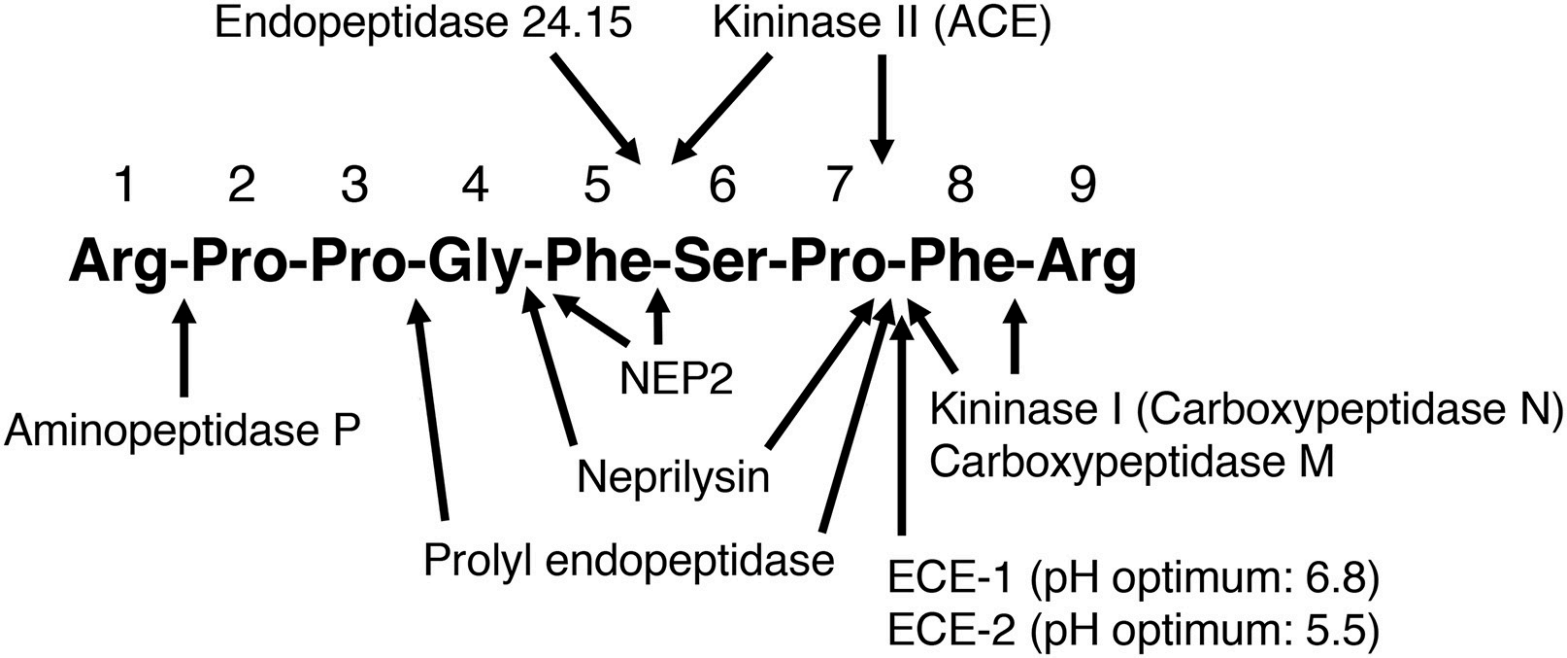
Sites of cleavage of bradykinin by different enzymes. ACE, angiotensin converting enzyme; ECE-1, endothelin converting enzyme-1; ECE-2, endothelin converting enzyme-2; NEP2, neprilysin homolog membrane metalloendopeptidase-like 1. (2–5, 7, 25–28, 30, 32–38).

Neprilysin, also known as neutral endopeptidase 24.11, common acute lymphoblastic leukemia antigen (CALLA), and cluster of differentiation cell surface molecule 10 (CD10), is a member of the neprilysin (M13) family of metallopeptidases. The neprilysin family also includes the neprilysin homolog membrane metalloendopeptidase-like 1 (NEP2) (22), endothelin converting enzymes 1 and 2 (ECE-1 and ECE-2), endothelin converting enzyme-like 1 (ECEL1), phosphate-regulating neutral endopeptidase (PHEX), and the KELL blood group glycoprotein (23, 24). Neprilysin and several other members of the neprilysin family of metallopeptidases degrade bradykinin (Table 2, Figure 1). Neprilysin is a predominantly membrane-bound zinc-dependent metallopeptidase with a broad tissue distribution, including the central nervous system, kidney, and vascular endothelium (39). Neprilysin is expressed at a low level on the membrane of mononuclear cells, and at higher levels by neutrophils, lymphocytes, and lymphoid progenitors (40, 41). A soluble form of neprilysin is found in blood plasma, cerebrospinal fluid, amniotic fluid, and seminal plasma. Neprilysin has a broad substrate selectivity (17), preferentially cleaving peptides on the amino side of the hydrophobic residues phenylalanine, leucine, and methionine (39, 42, 43).

**Table 2:** Kinetic parameters of bradykinin hydrolysis by different enzymes.

**Enzyme**	***K*_m_ (μmol/L)**	***k*_cat_ (min^−1^)**	***k*_cat_/*K*_m_ (min^−1^ μmol/L^−1^)**	**References**
ACE (kininase II)	0.18, 1	500, 600	500, 3667	(25, 26)
Neprilysin (neutral endopeptidase 24.11)	34, 92, 120	1500, 4771, 6364	40, 44, 69	(3, 26, 27)
NEP2	2	150	75	(3)
Aminopeptidase P	21, 76, 280	720, 1560, 2280	8, 21, 34,	(5, 28, 29)
Carboxypeptidase N (kininase I)	19	58	3	(30)
Carboxypeptidase M	16	147	9.2	(31)
Neutral endopeptidase 24.15	4.9	89	18	(32)
Endothelin converting enzyme-1^*^	340	1380	4.1	(33)
Endothelin converting enzyme-2^†^	27.4	348	12.7	(34)

*ACE, angiotensin converting enzyme; NEP2, neprilysin homolog membrane metalloendopeptidase-like 1*.

* *pH 6.5*;

† *pH 5.5*.

## The kallikrein kinin system

**Figure 2:**
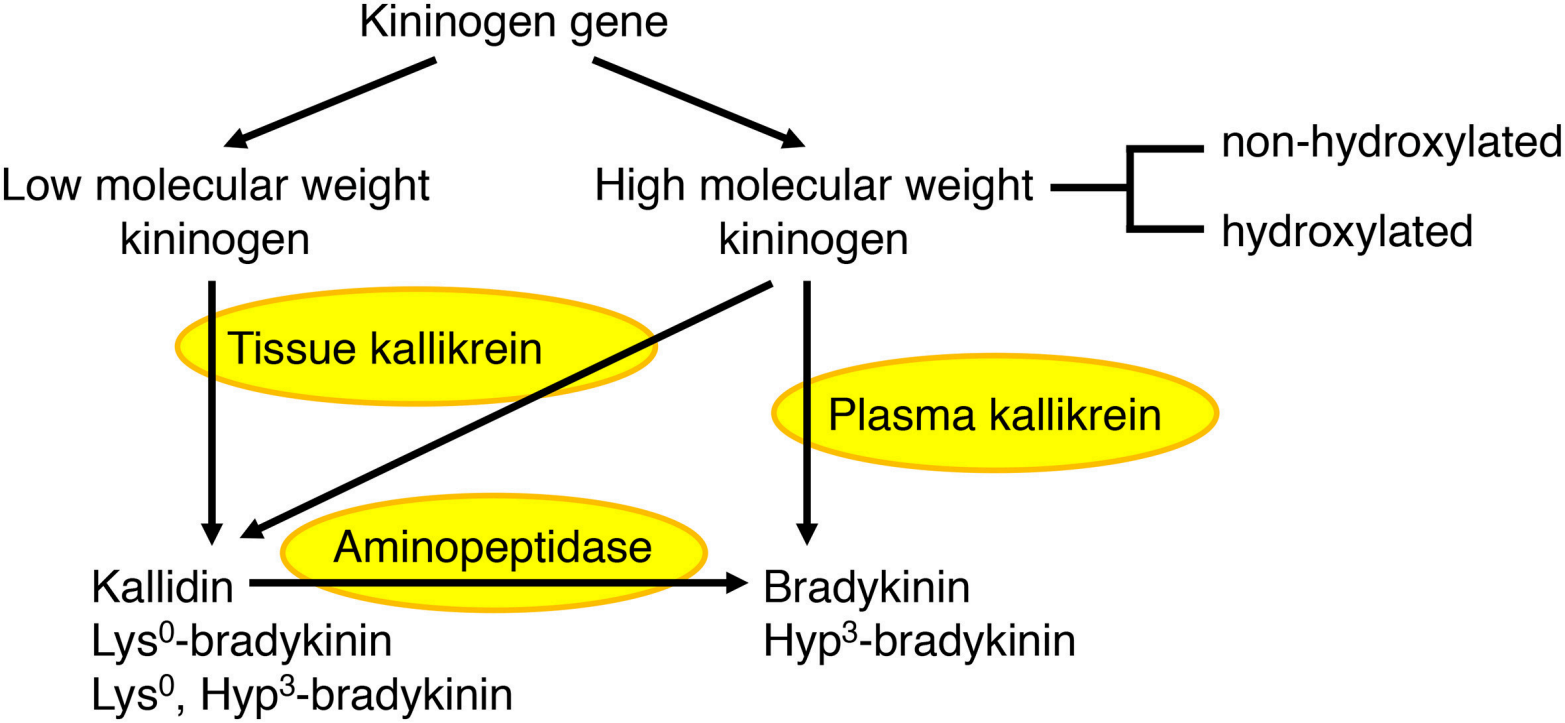
Formation of bradykinin and kallidin peptides. In humans, plasma kallikrein cleaves high molecular weight kininogen to produce bradykinin, whereas tissue kallikrein cleaves both high and low molecular weight kininogens to produce kallidin (Lys^0^-bradykinin). Bradykinin can also be generated by aminopeptidase-mediated cleavage of kallidin. A proportion of high molecular weight kininogen is hydroxylated on the third proline (Hyp^3^) of the bradykinin sequence, leading to the formation of both hydroxylated and non-hydroxylated bradykinin and kallidin peptides. In the rat, both plasma and tissue kallikrein produce bradykinin, which is not hydroxylated (44–47).

The kallikrein kinin system has been reviewed elsewhere (44–46). In humans, plasma kallikrein forms the nonapeptide bradykinin from high molecular weight kininogen, whereas tissue kallikrein forms the decapeptide kallidin (Lys^0^-bradykinin) from both high and low molecular weight kininogens (Figure 2). Bradykinin is also generated by aminopeptidase-mediated cleavage of kallidin. A proportion of high molecular weight kininogen is hydroxylated on the third proline of the bradykinin sequence, leading to the formation of both hydroxylated and non-hydroxylated bradykinin and kallidin peptides. Hydroxylated and non-hydroxylated kinin peptides are of similar abundance (48–50), and hydroxylated kinins have similar biological activity to non-hydroxylated kinins (46). In the rat, both plasma and tissue kallikrein produce bradykinin, which is not hydroxylated (47).

There are two types of kinin receptor, the type 1 (B_1_) receptor and the type 2 (B_2_) receptor. The B_2_ receptor normally predominates, whereas B_1_ receptors are induced by tissue injury. Bradykinin and kallidin are more potent on the B_2_ receptor, whereas the carboxypeptidase N (kininase I) metabolites bradykinin-(1-8) and Lys^0^-bradykinin-(1-8) are also bioactive and more potent on B_1_ receptors (46). Kinin peptides have a broad spectrum of activities that include the regulation of blood vessel tone and renal function, and protection from ischemia reperfusion injury (45). However, kinins also participate in inflammation, producing vasodilatation, increased vascular permeability, neutrophil chemotaxis and pain (45).

### Tissue specific regulation of kinin levels

The kallikrein kinin system is primarily a tissue-based system, with tissue kinin levels much higher than blood kinin levels in both humans and in rats (47–49). Evidence for the tissue-specific regulation of the kallikrein kinin system is the marked variation in kinin levels between different tissues of the rat (47). Kinin peptide levels are also higher in atrial tissue than blood of humans (48, 49). Moreover, bradykinin peptide levels are higher than kallidin peptide levels in blood and atrial tissue of humans, whereas kallidin peptide levels are much higher than bradykinin peptide levels in urine (48, 49). Many different enzymes cleave bradykinin and may participate in its metabolism (Figure 1), and peptidase activity plays a major role in the tissue-specific regulation of bradykinin levels (51).

## Role of neprilysin in bradykinin metabolism

**Figure 3:**
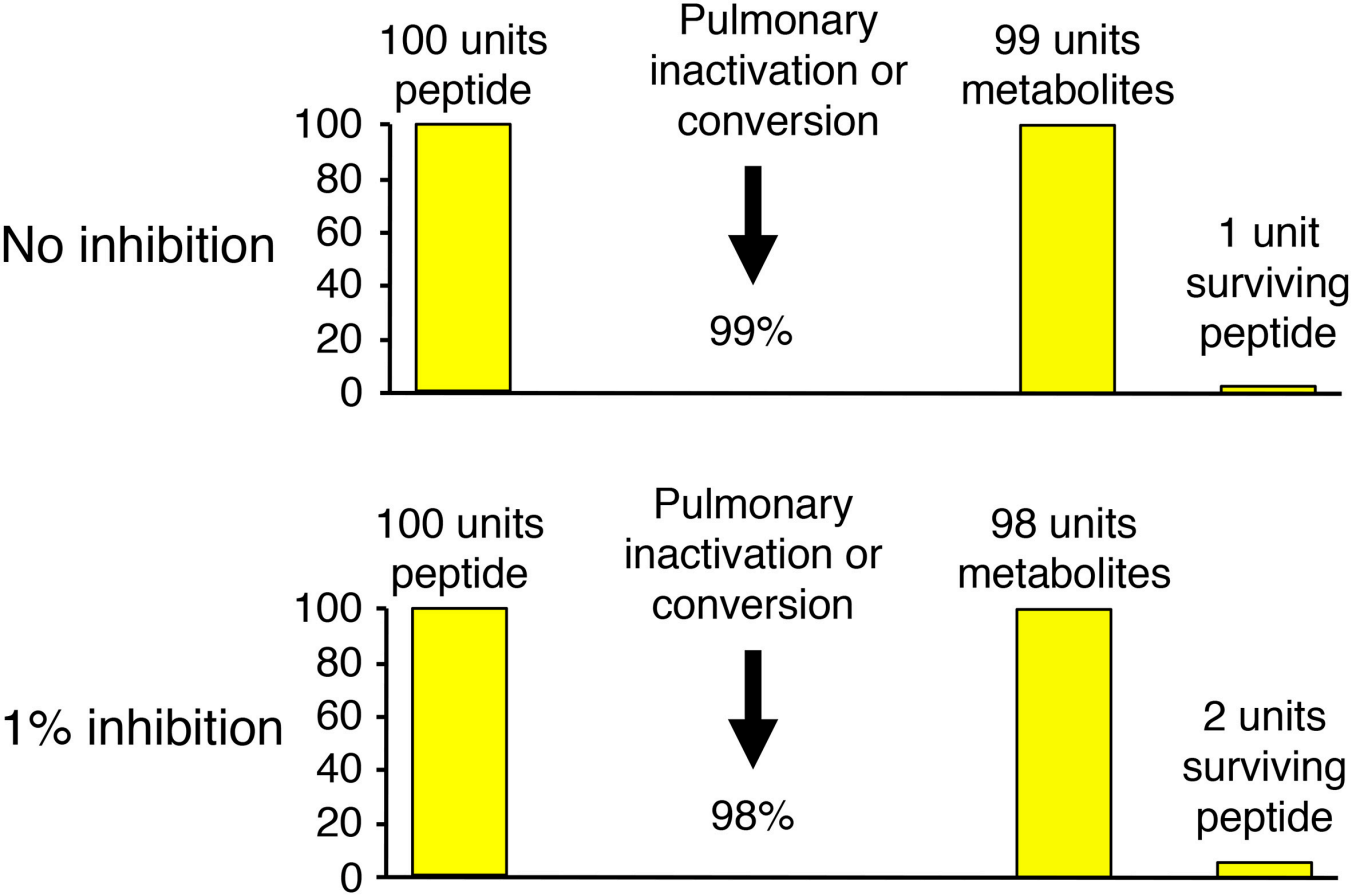
Illustration of how the effect of inhibition of an enzyme on bradykinin levels depends not only on the specific enzyme's contribution to bradykinin metabolism, relative to other enzymes, but also the baseline degradation rate for bradykinin. Pulmonary inactivation of bradykinin is approximately 99% (51). Thus, 1% inhibition of pulmonary inactivation will double the amount of bradykinin surviving pulmonary degradation, and may therefore double the level of bradykinin in arterial blood.

Several different experimental approaches have been used to study the role of neprilysin in bradykinin metabolism. These include study of the effects of neprilysin gene (*MME*) deletion and mutation, study of the effects of neprilysin inhibitor administration on physiological bradykinin levels, and study of the metabolism of exogenous (supra-physiological) bradykinin levels and the effect of neprilysin and other peptidase inhibitors on the metabolism of exogenous bradykinin. The effect of inhibition of an enzyme on bradykinin levels depends not only on the specific enzyme's contribution to bradykinin metabolism, relative to other enzymes, but also the baseline degradation rate for bradykinin. This is best illustrated by bradykinin metabolism by the pulmonary circulation, where bradykinin is degraded with approximately 99% efficiency (51). Thus, 1% inhibition of pulmonary inactivation will double the amount of bradykinin surviving pulmonary degradation, and may therefore double the level of bradykinin in arterial blood (Figure 3).

### Neprilysin gene knockout in mice

Neprilysin gene knockout in mice causes increased basal vascular permeability, hypotension and reduced heart weight/body weight ratio (52). The reduced heart weight/body weight ratio was attributed to the lower blood pressure. The vascular permeability, but not hypotension, was reversed by administration of recombinant neprilysin, and also by separate administration of SR140333, a substance P (NK1) receptor antagonist, and the bradykinin B_2_ receptor antagonist icatibant. The increased basal vascular permeability of neprilysin gene knock out was reproduced by administration of the neprilysin inhibitors thiorphan and phosphoramidon (Table 1) to wild-type C57BL/6 mice. These observations indicate an important role for neprilysin in the control of bradykinin- and substance P-mediated regulation of vascular permeability and blood pressure in the mouse. Bradykinin stimulates substance P release from sensory neurons (53), and neprilysin degrades both peptides (54), thereby providing an explanation why neprilysin gene knockout or neprilysin inhibition could increase both bradykinin and substance P levels, and why either a substance P receptor or bradykinin B_2_ receptor antagonist was able to prevent the increased vascular permeability.

Other consequences of neprilysin gene knockout in the mouse include hyperalgesia and increased susceptibility to inflammation (55, 56), enhanced lethality in response to endotoxin-induced shock (57), shortened ventilatory expiratory time in response to a hypoxic stimulus (58), and improved learning and memory (59). However, apart from hyperalgesia, which was reduced by icatibant (55), the relevance of these consequences of neprilysin gene knockout to bradykinin is unknown.

### Neprilysin gene deletion and neprilysin gene mutation in humans

In contrast to the effects of neprilysin gene knockout in the mouse, five women with total neprilysin deficiency due to homozygous truncating mutations of the neprilysin gene had no reported phenotype, although the absence of neprilysin induced an alloimmunization process against neprilysin present in fetal cells, leading to membranous glomerulopathy in their infants (60).

Loss–of-function and missense mutations in the neprilysin gene are associated with polyneuropathy, and also with decreased tissue availability of neprilysin and reduced neprilysin enzymatic activity (61–63), although the relevance of the polyneuropathy to bradykinin is unknown. However, the association of the rs989692 variant of the neprilysin gene with ACE inhibitor-associated angioedema (64) is evidence for a role for neprilysin in the regulation of bradykinin levels in humans.

### Effect of neprilysin inhibition on physiological bradykinin levels

We examined the effect of the neprilysin inhibitor ecadotril (acetorphan, an orally active prodrug of (*S*)-thiorphan) on bradykinin levels in Sprague Dawley rats (65). Ecadotril administration produced dose-related occupancy of renal neprilysin, as determined by binding of the neprilysin radioligand ^125^I-RB104 to kidney sections, and increased total neprilysin levels in plasma, similar to the induction of plasma ACE levels by ACE inhibitor therapy (66, 67). Ecadotril administration produced diuresis, natriuresis and increased urinary excretion of cyclic GMP and bradykinin, indicating a role for neprilysin in bradykinin degradation in renal tubules and/or in urine. However, ecadotril administration did not increase bradykinin levels in blood or renal tissue, although the ACE inhibitor perindopril increased bradykinin levels in both blood and kidney. Ecadotril did, however, increase cardiac bradykinin levels by approximately 2-fold; although the increase in cardiac bradykinin levels did not achieve statistical significance, ecadotril produced a statistically significant reduction in the bradykinin-(1-7)/bradykinin-(1-9) ratio in the heart, consistent with reduced formation of bradykinin-(1-7) by neprilysin-mediated cleavage of bradykinin (Figure 1), and indicating a role for neprilysin in bradykinin metabolism in the heart.

Neprilysin inhibition also increased urinary bradykinin excretion in deoxycorticosterone acetate (DOCA)-salt hypertensive rats, spontaneously hypertensive rats and renovascular hypertensive rats (68). Together, these studies indicate a role for neprilysin in bradykinin metabolism in renal tubules and/or urine, and also in the heart.

### Role of neprilysin in the metabolism of supra-physiological bradykinin levels

**Figure 4:**
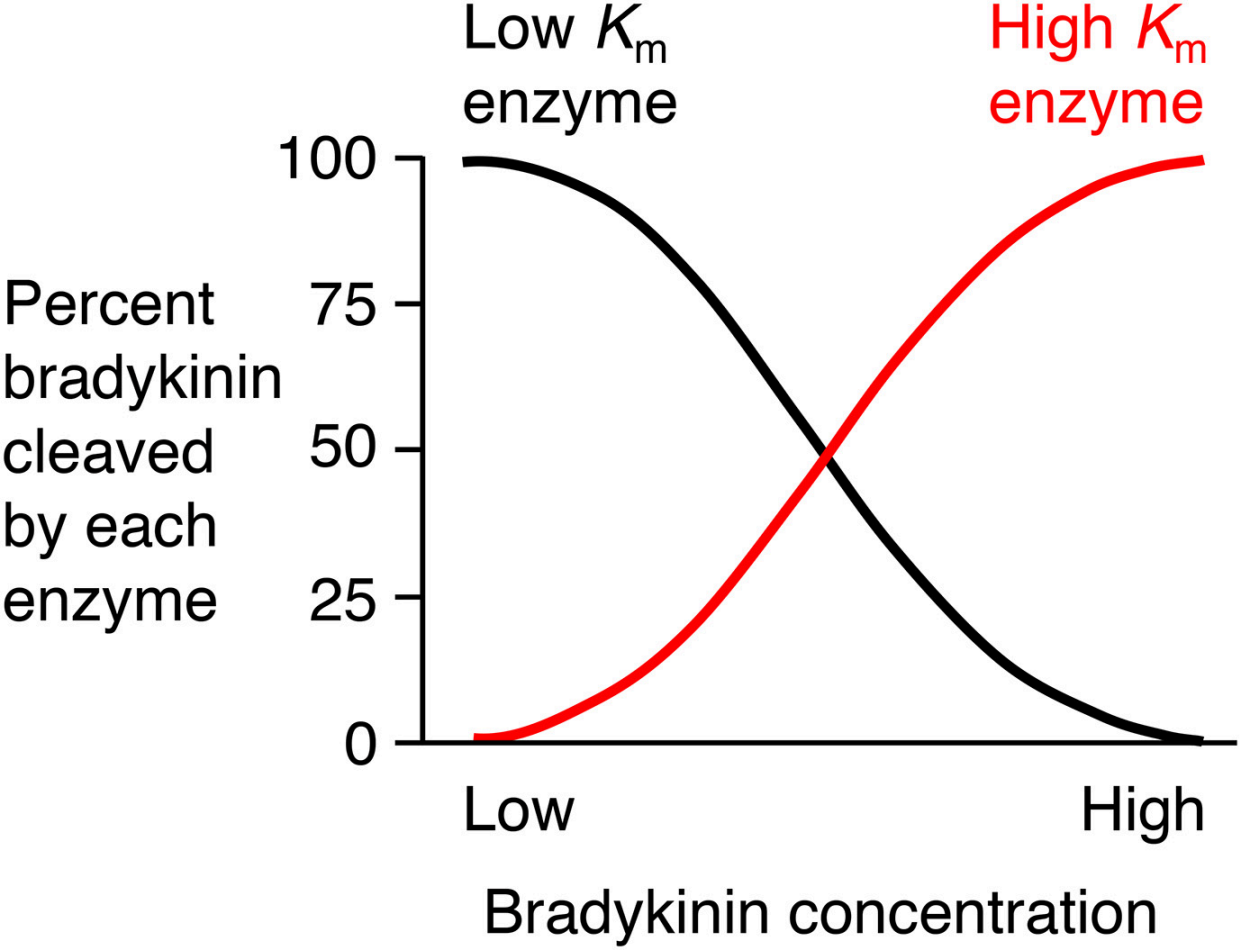
Relative contributions of low *K*_m_ (Michaelis constant) enzyme and high *K*_m_ enzyme to bradykinin degradation by a mixture of low and high *K*_m_ enzymes. An enzyme's contribution to bradykinin degradation, and the effect of inhibition of that enzyme on bradykinin degradation, depends not only on the concentration of the enzyme but also on the bradykinin concentration and the *K*_m_ of the enzyme for bradykinin degradation. Thus, depending on an enzyme's abundance and *k*_cat_ (turnover number), an enzyme with a low *K*_m_, such as ACE, may have a predominant role in bradykinin metabolism when bradykinin levels are low, whereas an enzyme with a higher *K*_m_, such as carboxypeptidase M or N, may play a more dominant role when bradykinin levels are high. Based on data reported by Kuoppala et al. (35).

There is need for caution in the interpretation of studies of the degradation of exogenously administered bradykinin where bradykinin levels may be considerably higher than physiological levels. An enzyme's contribution to bradykinin degradation, and the effect of inhibition of that enzyme on bradykinin degradation, depends not only on the concentration of the enzyme but also on the bradykinin concentration and the *K*_m_ (Michaelis constant) of the enzyme for bradykinin degradation (Figure 4). Thus, depending on an enzyme's abundance and *k*_cat_ (turnover number), an enzyme with a low *K*_m_, such as angiotensin converting enzyme (ACE) (Table 2), may have a predominant role in bradykinin metabolism when bradykinin levels are low, whereas an enzyme with a higher *K*_m_, such as carboxypeptidase M or N, may play a more dominant role when bradykinin levels are high (35).

Two approaches have been used to investigate the metabolism of exogenously administered bradykinin, either examination of the metabolites of bradykinin, or comparison of the effects of different peptidase inhibitors on bradykinin metabolism. Bradykinin-(1-5) was the predominant bradykinin metabolite when bradykinin was infused into human subjects (69), indicative of cleavage by ACE. Moreover, ACE played a predominant role in bradykinin metabolism by human and rat plasma and serum (35, 70–75), with a lesser contribution by carboxypeptidase (kininase I) and aminopeptidase activities. However, carboxypeptidase, a peptidase with higher *K*_m_ than ACE (Table 2) played a greater role than ACE in bradykinin metabolism when human or rat serum was incubated with ≥μmol/L bradykinin concentrations (76, 77), thereby illustrating how higher bradykinin concentrations can lead to a greater contribution by an enzyme with higher *K*_m_ to bradykinin metabolism (35).

Another example where a higher concentration of bradykinin led to a greater contribution to bradykinin metabolism by a peptidase with higher *K*_m_ is the study of bradykinin metabolism by the isolated perfused rat mesenteric arterial bed (78). When bradykinin metabolism was assessed by recovery of bradykinin in the perfusate after injection of ~100 nmol bradykinin, carboxypeptidase inhibition, and to a lesser extent neprilysin inhibition, but not ACE inhibition, reduced bradykinin metabolism (78). These findings were supported by the greater role played by carboxypeptidase B than ACE in the degradation of μmol/L concentrations of bradykinin by mesenteric arterial perfusate (79). However, the opposite result was obtained when bradykinin metabolism was assessed by the vasodilator response of the isolated perfused rat mesenteric arterial bed to ~100 pmol bradykinin, whereby the vasodilator response was potentiated by ACE inhibition, but not by either carboxypeptidase or neprilysin inhibition (78).

ACE played a greater role than neprilysin in bradykinin metabolism by isolated human small resistance vessels (80). Additionally, ACE played a dominant role, with a lesser role for aminopeptidase P, carboxypeptidase, and neprilysin, in bradykinin metabolism by the rat pulmonary vascular bed (10, 51, 77, 81), the isolated perfused rat heart (82–86), and isolated porcine coronary arteries (87), and was the predominant kininase in coronary perfusate, with a lesser role for neprilysin and carboxypeptidase (79). ACE was also the dominant peptidase contributing to bradykinin metabolism by the isolated perfused rat kidney, without evidence for contribution by neprilysin, carboxypeptidase, or aminopeptidase P (88).

A key limitation of the studies of the role of neprilysin in bradykinin metabolism so far described is the failure to address how different peptidases may make different contributions to bradykinin metabolism in different tissue compartments. In support of a tissue compartment-specific role for neprilysin in bradykinin metabolism, studies of lung, cardiac and renal bush border membranes, and urine, demonstrated a contribution by neprilysin that was equal to (89), or greater than (89–91), the contribution of ACE. Further evidence for a tissue compartment-specific role for neprilysin in bradykinin degradation was the metabolism of a bolus of ^3^H-bradykinin by the isolated perfused rat heart, which showed a delayed release of ^3^H-bradykinin-(1-7) into the perfusate, consistent with ^3^H-bradykinin-(1-7) formation in the interstitial compartment of the heart by neprilysin-mediated cleavage of ^3^H-bradykinin (84).

## Role of kinins in mediating the effects of neprilysin inhibition

**Figure 5:**
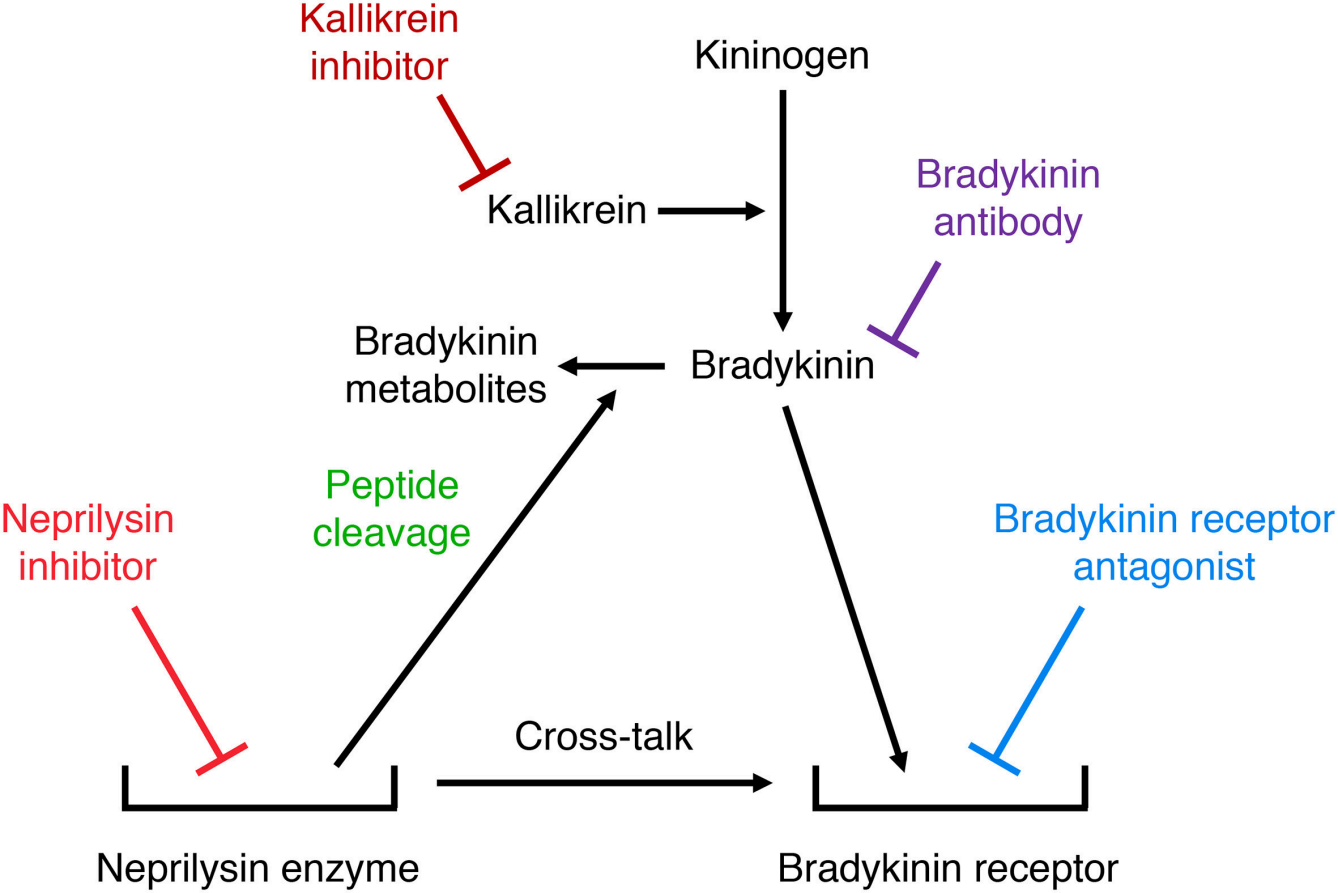
Illustration of two different mechanisms by which neprilysin inhibitors may potentiate bradykinin receptor-mediated actions. Firstly, neprilysin inhibitors may increase bradykinin receptor occupancy by inhibiting bradykinin degradation and increasing bradykinin levels in the vicinity of the receptor. Secondly, neprilysin inhibitors may promote cross-talk between the neprilysin-inhibitor complex and the bradykinin receptor. Bradykinin receptor antagonists, anti-bradykinin antibodies, and kallikrein inhibitors have different effects on these two mechanisms of neprilysin inhibitor-induced potentiation of bradykinin receptor-mediated actions. A bradykinin receptor antagonist that occupies the bradykinin receptor can prevent both mechanisms of neprilysin inhibitor-induced potentiation of bradykinin receptor-mediated actions. However, bradykinin antibodies that prevent bradykinin binding to its receptor by sequestering bradykinin, and kallikrein inhibitors that prevent bradykinin binding to its receptor by preventing its formation, do not impact on cross-talk between the neprilysin-inhibitor complex and the bradykinin receptor.

Many studies have used either bradykinin receptor antagonists, anti-bradykinin antibodies, or serine protease (kallikrein) inhibitors to demonstrate a role for bradykinin in mediating the effects of neprilysin inhibitors. Two different mechanisms may account for the potentiation of bradykinin receptor-mediated actions by neprilysin inhibitors (Figure 5). Firstly, neprilysin inhibitors may potentiate bradykinin receptor-mediated actions by inhibiting bradykinin degradation and increasing bradykinin levels in the vicinity of the receptor. Secondly, neprilysin inhibitors may potentiate bradykinin receptor-mediated actions by promoting cross-talk between the neprilysin-inhibitor complex and the bradykinin receptor (92), similar to the cross-talk between the ACE-inhibitor complex and the B_2_ receptor proposed to mediate ACE inhibitor-induced potentiation of bradykinin receptor-mediated effects (93). Bradykinin receptor antagonists, anti-bradykinin antibodies, and kallikrein inhibitors have different effects on these two mechanisms of neprilysin inhibitor-induced potentiation of bradykinin receptor-mediated actions. A bradykinin receptor antagonist that occupies the bradykinin receptor can prevent both mechanisms of neprilysin inhibitor-induced potentiation of bradykinin receptor-mediated actions. However, bradykinin antibodies that prevent bradykinin binding to its receptor by sequestering bradykinin, and kallikrein inhibitors that prevent bradykinin binding to its receptor by preventing its formation, do not impact on cross-talk between the neprilysin-inhibitor complex and the bradykinin receptor. Therefore, prevention of the effects of neprilysin inhibition by bradykinin antibodies or kallikrein inhibitors indicates that these effects are mediated by increased bradykinin levels consequent to inhibition of neprilysin-mediated bradykinin degradation, and not by cross-talk between the neprilysin-inhibitor complex and the bradykinin receptor.

### Role of kinins in mediating the renal effects of neprilysin inhibition

Icatibant prevented the diuretic and natriuretic effects of neprilysin inhibition in normal Sprague Dawley rats (94–96). Bradykinin receptor antagonism also prevented the neprilysin inhibitor-induced potentiation of atrial natriuretic peptide-induced diuresis and natriuresis in rats (97) and in chronic caval dogs (98). Moreover, anti-bradykinin antibodies prevented neprilysin inhibitor-induced potentiation of diuresis, natriuresis and increase in urinary cyclic GMP excretion in volume-expanded rats (99). However, in contrast to studies in normal Sprague Dawley rats, icatibant did not prevent the natriuretic effects of neprilysin inhibition in DOCA-salt hypertensive rats (96, 100), which suggests that the effects of neprilysin inhibition in DOCA-salt hypertensive rats are primarily mediated by increased natriuretic peptide levels consequent to inhibition of natriuretic peptide metabolism.

### Role of kinins in mediating the cardiac effects of neprilysin inhibition

Icatibant prevented neprilysin inhibitor-induced reduction in ischemia-reperfusion injury in the rat heart (101), and neprilysin inhibitor-induced potentiation of pre-conditioning-induced reduction in infarct size in the rabbit heart (102). In addition, icatibant prevented neprilysin inhibitor-induced reversal of isoproterenol-induced myocardial hypoperfusion in the rat (103), and neprilysin inhibitor-induced nitric oxide production by isolated canine coronary microvessels (104). Neprilysin inhibitor-induced nitric oxide production by isolated canine coronary microvessels was also prevented by the serine protease (kallikrein) inhibitor dichloroisocoumarin (104).

## Omapatrilat and bradykinin

Despite the potential therapeutic benefits of increased natriuretic peptide and bradykinin levels, neprilysin inhibitor therapy has only modest efficacy in essential hypertension and heart failure, which might be due in part to the inhibition of neprilysin metabolism of the vasoconstrictors angiotensin II and endothelin 1, and the increased plasma angiotensin II, endothelin 1 and noradrenaline levels that accompany neprilysin inhibitor therapy (17). Therefore, to prevent the renin angiotensin system from countering the therapeutic benefits of neprilysin inhibition, neprilysin inhibitor therapy was combined with inhibition of the renin angiotensin system, leading to the development of omapatrilat. Omapatrilat is a single molecule that inhibits both neprilysin and ACE (Table 1). Additionally, omapatrilat inhibits aminopeptidase P, NEP2, and ECE-1 (Table 1). There is currently no information on the effects of omapatrilat on bradykinin levels. However, given that both neprilysin and ACE degrade bradykinin, one would predict higher bradykinin levels with omapatrilat than ACE inhibitor therapy, which no doubt accounts for the higher incidence of angioedema with omapatrilat therapy. The incidence of angioedema was higher for omapatrilat therapy (2.17%) than for enalapril therapy (0.68%) in hypertensive patients (105), and omapatrilat failed to achieve regulatory approval because of the angioedema incidence. However, the incidence of angioedema was lower in patients with HFrEF, without statistically significant difference between omapatrilat therapy (0.8%) and enalapril therapy (0.5%) (106).

The potential consequences of combined neprilysin and ACE inhibition were examined in the rat tracheal plasma extravasation assay (Table 3). Whereas neither ecadotril, sufficient to produce >90% inhibition of renal neprilysin, nor lisinopril, sufficient to produce 83% inhibition of lung ACE, produced plasma extravasation, their combination produced plasma extravasation, suggesting that their combination increased bradykinin (and substance P) levels sufficient to cause extravasation. It is also possible that omapatrilat-induced inhibition of aminopeptidase P, NEP2, and ECE-1 (Table 1) contributed to increased bradykinin (and substance P) levels and the plasma extravasation observed in rats, and angioedema in patients administered this therapy.

**Table 3:** Effects of combined renin angiotensin system and neprilysin inhibition on tracheal plasma extravasation in the rat.

**Compound**	**Tracheal plasma extravasation**	**APP inhibition *K*_i_ or IC_50_ (nmol/L)**	**References**
**ACE AND NEPRILYSIN INHIBITION**			
Ecadotril (99% neprilysin & 23% ACE inhibition)	No	No	(5, 107)
Lisinopril (83% ACE inhibition)	No	No	(10, 11, 107, 108)
Ecadotril & Lisinopril	Yes	No	(5, 107)
Omapatrilat (>90% ACE & 53% neprilysin inhibition)	Yes	194, 250, 260	(10, 11, 13)
**ARB AND NEPRILYSIN INHIBITION**			
Valsartan (100 mg/kg)	No	No	(11)
Candoxatril (100 mg/kg)	No	No	(11)
Valsartan & candoxatril	No	No	(11)
Omapatrilat (0.3 mg/kg)	Yes^*^	194, 250, 260	(10, 11, 13)

* *Plasma extravasation caused by omapatrilat was prevented by prior icatibant administration (11). ACE, angiotensin converting enzyme; APP, aminopeptidase P; ARB, type 1 angiotensin II receptor blocker*.

## LCZ696 and bradykinin

There is currently no information on the effects of LCZ696, sacubitril or LBQ657 on bradykinin levels. However, several lines of evidence indicate a role for bradykinin in the therapeutic benefits of LCZ696 therapy, and also the angioedema associated with this therapy. Whereas ARBs produce angioedema with an incidence approximately half that of ACE inhibitor therapy in patients without heart failure (109, 110), LCZ696 produces angioedema with an incidence at least equal to that of ACE inhibitor therapy (111). In the Prospective comparison of Angiotensin Receptor-neprilysin inhibitor with Angiotensin converting enzyme inhibitor to Determine Impact on Global Mortality and morbidity in Heart Failure (PARADIGM-HF) study of patients with HFrEF, angioedema was confirmed in 0.45% of patients receiving LCZ696 therapy and 0.24% of patients receiving enalapril therapy (111), a numerical difference that was not statistically significant (*P* = 0.13). However, the protocol of the PARADIGM–HF study might have resulted in a lower incidence of angioedema in the trial population than might occur in patients naive to LCZ696 therapy. The exclusion criteria for the PARADIGM-HF study included a history of angioedema during treatment with an ACE inhibitor or ARB, and 78 and 22% of participants, respectively, were previously treated with an ACE inhibitor or ARB. Additionally, the study involved a run-in period before randomization during which participants received at least 2 weeks of enalapril therapy, followed by 4–6 weeks of LCZ696 therapy.

### ARBs increase bradykinin levels

Losartan increases bradykinin levels approximately 2-fold in arterial blood of patients with hypertension (50), similar to the increase seen with ACE inhibition (112, 113). Eprosartan produced a similar increase in bradykinin levels in the same patients, although the increase did not achieve statistical significance (50). By contrast, neither losartan nor valsartan increased bradykinin levels in rats (114, 115). There are conflicting data on the role of bradykinin in mediating the effects of ARBs. Both animal and human studies implicate kinin peptides and/or the B_2_ receptor in the actions of ARBs, possibly mediated by AT_2_ receptor stimulation by the increased angiotensin II levels that accompany ARB therapy (116–124). However, in contrast to the attenuation of the hypotensive effects of ACE inhibition by concomitant icatibant administration (100 μg/kg/h iv for 1 h) in sodium-deplete normotensive and hypertensive subjects (125), and at a higher dose (10 mg infused iv over 15 min) in sodium replete normotensive subjects (126), a lower dose of icatibant (18 μg/kg/h iv for 6 h) did not attenuate the hypotensive effects of either acute or chronic administration of valsartan in sodium-deplete normotensive and hypertensive subjects (127).

### LBQ657 inhibits not only neprilysin but also ACE, NEP2, and ECE-2

In contrast to the plasma transudation seen with combined neprilysin and ACE inhibition in the rat tracheal plasma transudation model (Table 3), no transudation occurred when candoxatril was combined with valsartan (11), suggesting that combined neprilysin inhibitor and ARB therapy may cause less increase in bradykinin levels than combined neprilysin and ACE inhibition. However, LBQ657 may inhibit enzymes other than neprilysin that degrade bradykinin (Table 1). Ksander et al. reported that 10 μmol/L LBQ657 produced <50% inhibition of ACE (14). Moreover, based on information provided by Novartis Europharm Ltd, the Committee for Medicinal Products for Human Use (CHMP) of the European Medicines Agency reports that LBQ657 inhibits not only ACE but also NEP2 and ECE-2 (15). It is notable that peak LBQ657 concentrations approximated 37 μmol/L in healthy subjects following 400 mg/day LCZ696, and trough concentrations of LBQ657 (24 h post 400 mg LCZ696) were 4.8 μmol/L. The trough LBQ657 concentration (4.8 μmol/L) is ~2,000 times the K_*i*_ of 2.3 nmol/L for neprilysin inhibition by LBQ657 (16), and the peak LBQ657 concentration is correspondingly higher. Thus, recommended doses of LCZ696 (400 mg/day) may produce LBQ657 concentrations sufficient to inhibit ACE and contribute to increased bradykinin levels, given that, as discussed earlier, as little as 1% inhibition of pulmonary inactivation of bradykinin can double bradykinin levels (Figure 3). Furthermore, NEP2 has a much lower *K*_m_ for bradykinin than NEP (Table 2) and NEP2 inhibition by LBQ657 may also increase bradykinin levels. LBQ657-mediated inhibition of ECE-2 is unlikely to contribute to increased bradykinin levels because ECE-2 is relatively inactive at physiological pH (7, 34).

**Figure 6:**
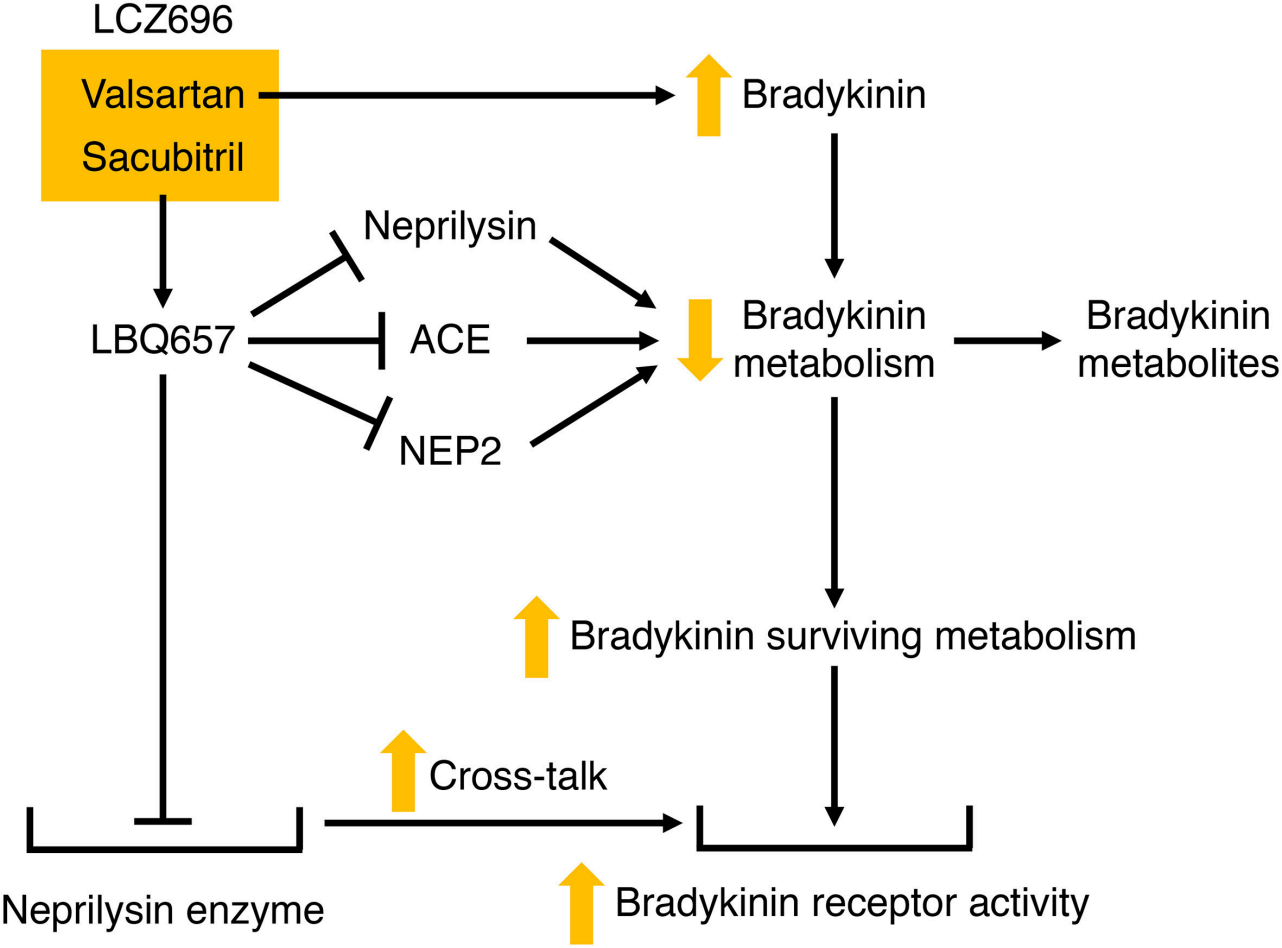
Potential mechanisms by which LCZ696 may potentiate bradykinin receptor-mediated actions. Valsartan may increase bradykinin levels, and LBQ657 may also increase bradykinin levels by inhibiting bradykinin degradation by neprilysin, and possibly angiotensin converting enzyme (ACE) and neprilysin homolog membrane metalloendopeptidase-like 1 (NEP2). In addition, LBQ657 may potentiate bradykinin receptor-mediated actions by cross-talk between the LBQ657-inhibitor complex and the bradykinin receptor.

LCZ696 therapy may therefore potentiate bradykinin-mediated actions by several mechanisms (Figure 6). These include the increase in bradykinin levels with ARB therapy (50), the increase in bradykinin levels consequent to LBQ657-mediated inhibition of neprilysin and possibly ACE and NEP2, and cross-talk between the neprilysin-LBQ657 complex and the bradykinin receptor. Bradykinin-mediated actions will likely contribute to not only the renal and cardioprotective effects but also the angioedema associated with LCZ696 therapy. Given that heart failure is associated with suppression of the kallikrein kinin system (48, 128), and resistance to kinin-mediated cutaneous transudation (129), there is concern that LCZ696 therapy for conditions such as hypertension may be associated with a higher angioedema incidence than observed in patients with HFrEF.

## Summary

Tissue levels of bradykinin are higher than circulating levels and the contribution of neprilysin to bradykinin degradation is specific to the tissue and the tissue compartment. Bradykinin is a likely contributor to the therapeutic benefits of neprilysin inhibitor therapy, particularly the renal and cardioprotective effects. However, bradykinin is also an important contributor to angioedema that may result from peptidase inhibitor therapy, including neprilysin inhibitor therapy, particularly when neprilysin inhibition is combined with ACE inhibitor therapy. LBQ657 inhibits not only neprilysin but also ACE, NEP2, and ECE-2. Although angioedema incidence was acceptable, and similar for LCZ696 and enalapril therapy in HFrEF patients, it remains to be seen whether LCZ696 therapy for other conditions such as hypertension is also accompanied by an acceptable incidence of angioedema.

## Author contributions

The author confirms being the sole contributor of this work and has approved it for publication.

### Conflict of interest statement

The author declares received research funding from Servier Laboratories, Bayer Pharmaceutical Company, Solvay Pharmaceutical Company and Novartis Institutes for Biomedical Sciences, Inc.
